# Synthesis and redetermination of the crystal structure of salicyl­aldehyde *N*(4)-morpholino­thio­semi­carbazone

**DOI:** 10.1107/S2056989019011812

**Published:** 2019-08-30

**Authors:** Dang Tran Buu, Vu Duong Ba, Minh Khoi Nguyen Hoang, Trung Vu Quoc, Linh Duong Khanh, Yen Oanh Doan Thi, Luc Van Meervelt

**Affiliations:** a Faculty of Chemistry, Ho Chi Minh City University of Education, 280 An Duong Vuong Street, District No. 5, Ho Chi Minh City, Vietnam; b Vietnam National University, Ho Chi Minh City High School for the Gifted, 153 Nguyen Chi Thanh, District 5, Ho Chi Minh City, Vietnam; c Faculty of Chemistry, Hanoi National University of Education, 136 Xuan Thuy, Cau Giay, Hanoi, Vietnam; d Publishing House for Science and Technology, 18 Hoang Quoc Viet, Cau Giay, Hanoi, Vietnam; e Department of Chemistry, KU Leuven, Biomolecular Architecture, Celestijnenlaan 200F, Leuven (Heverlee), B-3001, Belgium

**Keywords:** crystal structure, thio­semicarbazone, hydrogen bonding, Hirshfeld analysis

## Abstract

The structure of the title compound (systematic name: *N*-{[(2-hy­droxy­phen­yl)methyl­idene]amino}­morpholine-4-carbo­thio­amide), C_12_H_15_N_3_O_2_S, was prev­iously determined (Koo *et al.*, 1977 ▸) using multiple-film equi-inclination Weissenberg data, but has been redetermined with higher precision to explore its conformation and the hydrogen-bonding patterns and supra­molecular inter­actions. The mol­ecular structure shows intra­molecular O—H⋯N and C—H⋯S inter­actions. The configuration of the C=N bond is *E*. The mol­ecule is slightly twisted about the central N—N bond. The best planes through the phenyl ring and the morpholino ring make an angle of 43.44 (17)°. In the crystal, the mol­ecules are connected into chains by N—H⋯O and C—H⋯O hydrogen bonds, which combine to generate sheets lying parallel to (002). The most prominent contribution to the surface contacts are H⋯H contacts (51.6%), as concluded from a Hirshfeld surface analysis.

## Abstract 2

In the crystalline state, salicyl­aldehyde *N*(4)-morpholino­thio­semicarbazone forms sheets parallel to (002) and consisting of two parallel chains running in the *a*-axis direction and formed by N—H⋯O and C—H⋯O hydrogen bonds.

## Chemical context   

For many years, scientific studies on cancer have attracted a lot of attention, especially in the field of anti­tumor drugs. Cisplatin is well known as an effective therapy to prohibit the proliferation of tumor cells (Berners-Price, 2011 ▸). However, this drug has some unforeseen side effects with detrimental effects on the patient’s health (Lévi *et al.*, 2000 ▸; Go & Adjei, 1999 ▸; Harbour *et al.*, 1996 ▸). In a search for anti­tumour drugs with fewer harmful side effects, thio­semicarbazides were examined since this organic class of thio­urea derivatives was known to possess a diversity of biological activities such as anti­tumoral, anti­bacterial, and anti­fungal activities owing to presence of the N—N—C=S system (Dilović *et al.*, 2008 ▸; Liberta & West, 1992 ▸). Many mechanisms have been advanced to probe the role of this conjugated system. In general, thio­semicarbazones can bind to nucleotides of tumour cells by the nitro­gen and sulfur atoms, which prevents the distorted DNA from translation and encryption for their growth (Dilović *et al.*, 2008 ▸).

Thio­semicarbazones are synthesized by the condensation between an aldehyde or ketone and an *N*(4)-substituted thio­semicarbazide. Many reports have demonstrated that *N*(4)-aromatic or heterocyclic substituted thio­semicarbazides are biologically more active than thio­semicarbazones without substituted groups (Dilović *et al.*, 2008 ▸; Chen *et al.*, 2004 ▸; Shi *et al.*, 2009 ▸). In addition, salicyl­aldehyde is a key compound in the synthesis of a variety of potential therapeutic products (Bindu *et al.*, 1998 ▸).
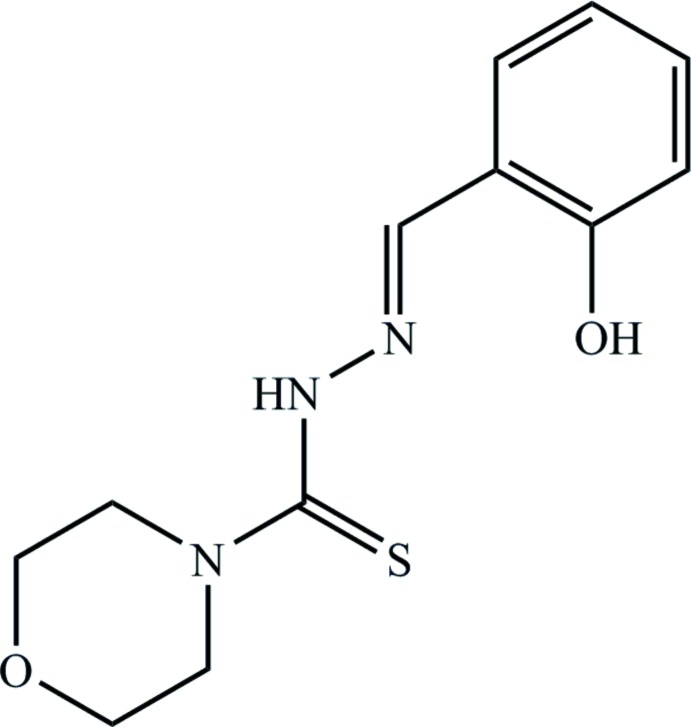


The crystal and mol­ecular structure of salicyl­aldehyde *N*(4)-morpholino­thio­semicarbazone was published previously (Koo *et al.*, 1977 ▸) based on multiple-film equi-inclination Weissenberg data using Cu *Kα* radiation and refined to an *R* value of 0.11. In this study, we present the synthesis of salicyl­aldehyde *N*(4)-morpholino­thio­semicarbazone (**3**) together with its structural characteristics and crystal structure redetermination using present-day technology.

## Structural commentary   

**Figure 1:**
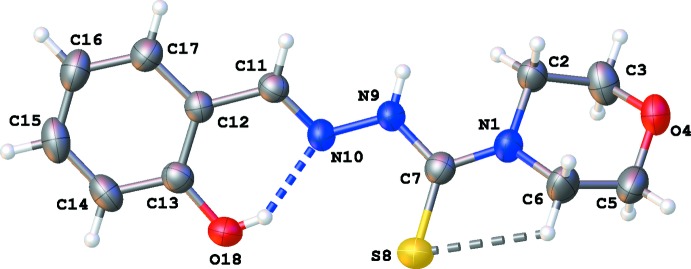
A view of the mol­ecular structure of (**3**), with atom labels and displacement ellipsoids drawn at the 50% probability level. H atoms are shown as small circles of arbitrary radii and the intra­molecular O—H⋯N and C—H⋯S inter­actions, respectively, by blue and grey dashed lines.

The title compound crystallizes in the ortho­rhom­bic space group *Pna*2_1_ with one mol­ecule in the asymmetric unit (Fig. 1 ▸). The N9—N10 and C11=N10 bond lengths are 1.371 (3) and 1.275 (3) Å, respectively (compared to 1.40 and 1.30 Å in the previous structure determination; Koo *et al.*, 1977 ▸). The configuration of the C11=N10 bond is *E* [the N9—N10—C11—C12 torsion angle is −179.9 (3)°], which gives rise to an intra­molecular O18—H18⋯N10 hydrogen bond with an 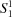
(6) graph-set motif (Table 1 ▸). The planes of the phenyl ring (r.m.s. deviation = 0.0020 Å) and the thio­semicarbazone function (N1/C7–C11; r.m.s. deviation = 0.0911 Å) make an angle of 16.26 (5)°. The mol­ecule is slightly twisted about the N9—N10 bond [torsion angle C7—N9—N10—C11 is 162.4 (3)°; +*ap* conformation].

The morpholino ring adopts a chair conformation [puckering parameters *Q* = 0.554 (3) Å, θ = 173.2 (3)° and φ = 214 (3)°] with the thio­semicarbazone function in an equatorial position. The plane of the phenyl ring forms a dihedral angle of 43.44 (17)° with the best plane through the morpholino ring. A second intra­molecular C6—H6*A*⋯S8 inter­action is observed (Table 1 ▸).

## Supra­molecular features   

**Figure 2:**
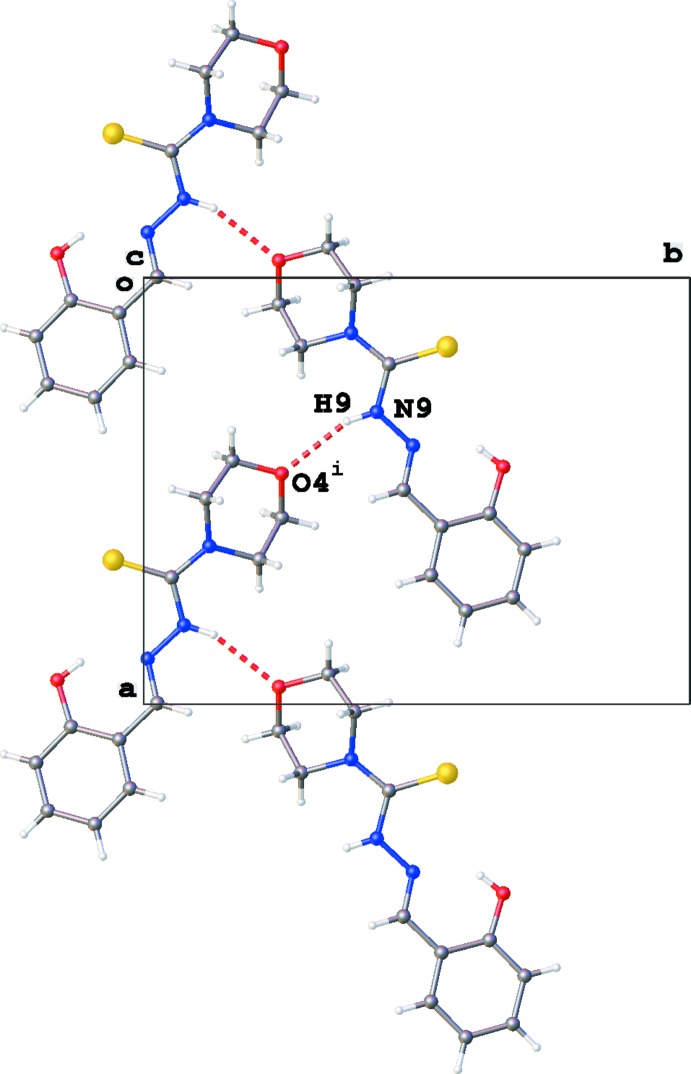
Partial crystal packing of (**3**), showing the N—H⋯O inter­actions (red dashed lines) resulting in chain formation in the *a*-axis direction [see Table 1 ▸; symmetry code: (i) *x* + 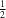, −*y* + 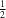, *z*].

**Figure 3:**
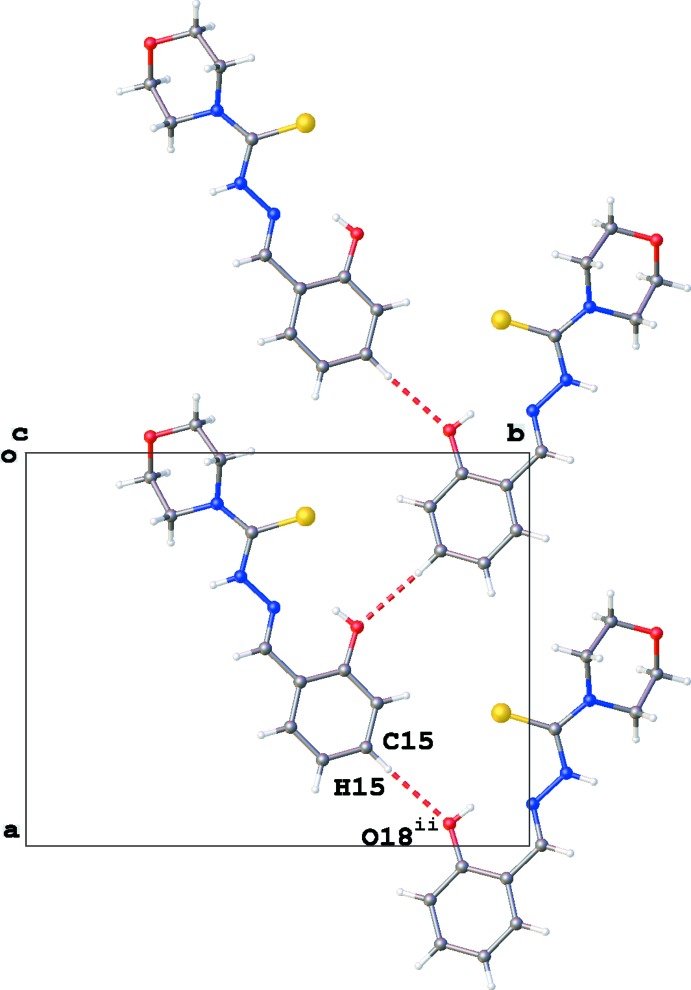
Partial crystal packing of (**3**), showing the C—H⋯O inter­actions (red dashed lines) resulting in chain formation in the *a*-direction [see Table 1 ▸; symmetry code: (ii) *x* + 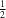, −*y* + 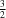, *z*].

The crystal packing of (**3**) is dominated by N9—H9⋯O4 hydrogen bonds (Table 1 ▸), resulting in the formation of chains of mol­ecules with graph-set motif 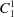
(7) propagating along the *a*-axis direction (Fig. 2 ▸). Furthermore, a second parallel chain of mol­ecules with graph-set motif 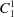
(5) running along the *a*-axis direction is formed by C15—H15⋯O18 inter­actions (Fig. 3 ▸). These two chain motifs combine to generate a sheet lying parallel to (002). No voids or π–π stackings are observed in the crystal packing of (**3**).

**Figure 4:**
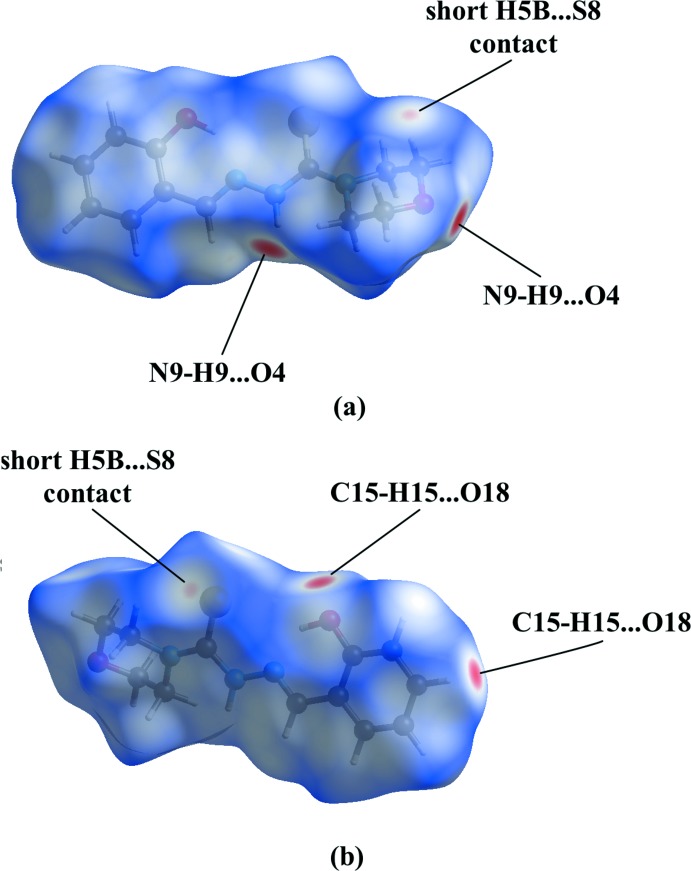
The Hirshfeld surface mapped over *d*_norm_ for (**3**) in the range −0.3153 to 1.2662 a.u.

**Figure 5:**
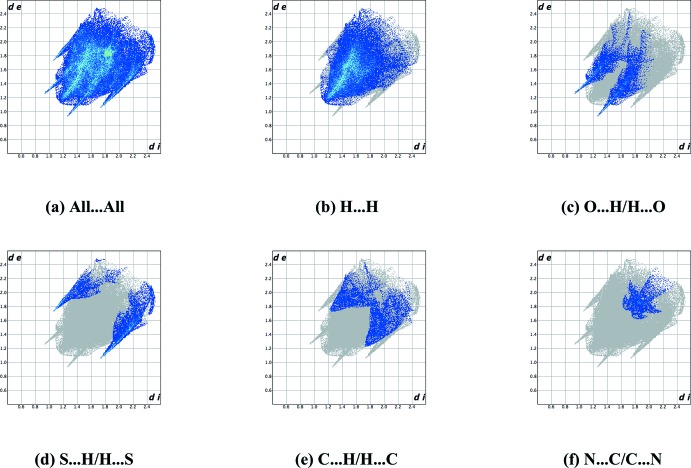
Full two-dimensional fingerprint plots for (**3**), showing (*a*) all inter­actions, and delineated into (*b*) H⋯H, (*c*) O⋯H/H⋯O, (*d*) S⋯H/H⋯S, (*e*) C⋯H/H⋯C and (*f*) N⋯C/C⋯N inter­actions. The *d*_i_ and *d*_e_ values are the closest inter­nal and external distances (in Å) from a given point on the Hirshfeld surface.

A Hirshfeld surface analysis (Spackman & Jayatilaka, 2009 ▸) and the associated two-dimensional fingerprint plots (McKinnon *et al.*, 2007 ▸) were performed in order to further investigate the supra­molecular network. The Hirshfeld surface calculated using *CrystalExplorer* (Turner *et al.*, 2017 ▸) and mapped over *d*_norm_ is given in Fig. 4 ▸. The bright-red spots near atoms O4 and N9 in Fig. 4 ▸ *a* refer to the N9—H9⋯O4 hydrogen bond, and near atoms C15 and O18 in Fig. 4 ▸ *b* to the C15—H15⋯O18 hydrogen bond. The faint-red spots near atoms C5 and S8 illustrate a short contact in the crystal packing of (**3**) (H5*B*⋯S8 = 2.913 Å). The fingerprint plots (Fig. 5 ▸) further indicate a major contribution by H⋯H contacts, corresponding to 51.6% of the two-dimensional fingerprint plot (Fig. 5 ▸ *b*). Significant contributions by reciprocal O⋯H/H⋯O (13.4%) and S⋯H/H⋯S (12.5%) contacts appear as two symmetrical spikes at *d*_e_ + *d*_i_ ≃ 2.2 and 2.8 Å, respectively (Fig. 5 ▸ *c*,*d*). Smaller contributions are from C⋯H/H⋯C (11.7%, Fig. 5 ▸ *e*), N⋯C/C⋯N (5.3%, Fig. 5 ▸ *f*), C⋯C (3.2%), N⋯H/H⋯N (1.6%), C⋯O/O⋯C (0.3%), C⋯S/S⋯C (0.3%) and O⋯O contacts (0.1%).

## Database survey   

**Figure 6:**
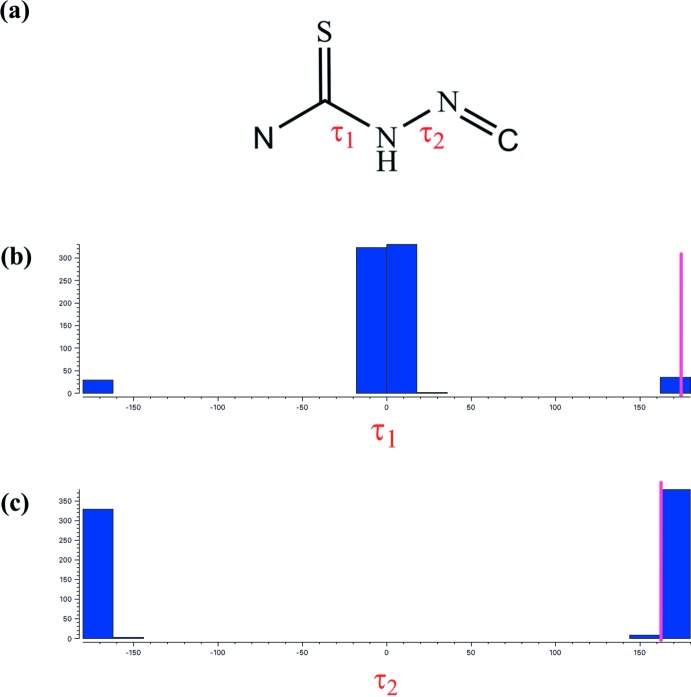
(*a*) Fragment used for a search in the CSD. (*b*),(*c*) Histograms of torsion angles τ_1_ and τ_2_. The vertical pink lines show the torsion angle observed in (**3**).

A search of the Cambridge Structural Database (CSD, Version 5.40, update of May 2019; Groom *et al.*, 2016 ▸) for the central N—C(=S)—NH—N=C moiety (Fig. 6 ▸ *a*) present in the title compound gave 583 hits. Fig. 6 ▸ *b*,*c* illustrate the histograms of the distribution of torsion angles τ_1_ and τ_2_. The histogram of τ_1_ shows a major preference for the −*sp*/+*sp* (or *cis*) conformation and a minor preference for the −*ap*/+*ap* (or *trans*) conformation. For torsion angle τ_2_, only one region is preferred: a narrow spread in the region −*ap*/+*ap* (or *trans*). For (**3**), the torsion angles τ_1_ and τ_2_ are both in the +*ap* region [τ_1_ = 173.8 (3) and τ_2_ = 162.4 (3)°].

The most similar compound present in the CSD is the 2-hy­droxy­naphthaldehyde-based thio­semicarbazone (refcode IDEQAM; Aneesrahman *et al.*, 2018 ▸). The asymmetric unit contains two mol­ecules (one morpholino ring shows disorder). The mean plane of the non-disordered morpholino ring makes an angle of 36.9 (7)° with the naphthalene ring system. The torsion angles τ_1_ [175.89 (15) and −175.97 (15)°] and τ_2_ [166.51 (16) and −174.99 (16)°] are similar to those observed for the title compound. An intra­molecular hydrogen bond similar to O18—H18⋯N10 is also observed.

## Synthesis and crystallization   

**Figure 7:**
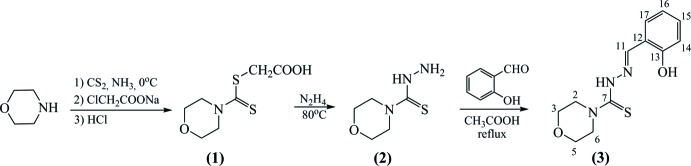
Reaction scheme for the synthesis of (**3**).

The reaction scheme for the synthesis of (**3**) is given in Fig. 7 ▸.

***Synthesis of 2-((morpholine-4-carbono­thio­yl)thio)­acetic acid (1)*** **:**

A mixture consisting of carbon di­sulfide (0.2 mol) and concentrated ammonia (25 mL) was stirred to form a homogeneous solution at 278 K. Then, morpholine (0.2 mol) was added dropwise to this solution. The yellow solid that separated from the solution was filtered off and immediately dissolved in deionized water (300 mL) at room temperature to generate a yellow solution. Sodium chloro­acetate (0.2 mol) was added to this solution and the reaction mixture maintained for 6 h at room temperature. The yellowish solution was acidified with concentrated hydro­chloric acid and the resulting white precipitate was filtered off and recrystallized from ethanol.

***Synthesis of N(4)-morpholino­thio­semicarbazide (2)*** **:**

A mixture composed of (**1**) (50 mmol), deionized water (10 mL) and hydrazine hydrate (25 mL) was refluxed for 30 minutes at 353 K. The white solid which precipitated from the transparent solution was filtered off and recrystallized from ethanol to give (**2**).

***Synthesis of salicyl­aldehyde N(4)-morpholino­thio­semicarbazone (3)*** **:**

After dissolving (**2**) in hot ethanol, the solution was added to an equivalent amount of salicyl­aldehyde. The final solution was refluxed at 353 K for 2 h in the presence of acetic acid as a catalyst. The resulting solution was gradually reduced in volume at room temperature overnight. The needle-shaped crystals that formed were filtered off and recrystallized from ethanol to give (**3**) in the form of transparent crystals (yield 60%), m.p. 461–463 K. FT–IR (cm^−1^): 3436 (O—H), 3279 (N—H), 1617 (C_Ar_—H), 1540 (C=N), 1061 (N—N), 1348 and 959 (C=S). ^1^H NMR [Bruker 500 MHz, *d_6_*-DMSO, δ (ppm), *J* (Hz)]: 3.67 (4H, *t*, H2 and H6); 3.92 (4H, *t*, H3 and H5); 6.90 (2H, *m*, H14 and H16); 7.28 (1H, *m*, *J = 7.5*, H15); 7.41 (1H, *d*, *J = 7.0*, H17); 8.47 (1H, *s*, H11); 11.49 (1H, *br*, N—H); 11.55 (1H, *br*, O—H). ^13^C NMR [Bruker 125 MHz, *d_6_*-DMSO, δ (ppm)]: 49.4 (C2 and C6), 66.2 (C3 and C5), 117.0 (C14), 118.9 (C12), 119.5 (C16), 130.4 (C17), 131.3 (C15), 146.9 (C13), 157.6 (C11), 180.1 (C7). UV–Vis (ethanol, nm): 200 (π→π*); 300 and 350 (n→π*).

## Refinement   

Crystal data, data collection and structure refinement details are summarized in Table 2 ▸. Both H atoms H9 and H18 were located from difference electron density maps and refined freely. The other H atoms were placed in idealized positions and included as riding contributions with *U*_iso_(H) values 1.2*U*_eq_ of the parent atoms, with C—H distances of 0.93 (aromatic, CH=N) and 0.97 Å (CH_2_). In the final cycles of refinement, 4 outliers were omitted.

## Supplementary Material

Crystal structure: contains datablock(s) I. DOI: 10.1107/S2056989019011812/mw2147sup1.cif

Structure factors: contains datablock(s) I. DOI: 10.1107/S2056989019011812/mw2147Isup2.hkl

Supporting information file. DOI: 10.1107/S2056989019011812/mw2147Isup3.cml

CCDC reference: 1949697

Additional supporting information:  crystallographic information; 3D view; checkCIF report

## Figures and Tables

**Table 1:** Hydrogen-bond geometry (Å, °)

*D*—H⋯*A*	*D*—H	H⋯*A*	*D*⋯*A*	*D*—H⋯*A*
N9—H9⋯O4^i^	0.86 (3)	2.33 (3)	3.141 (3)	157 (3)
O18—H18⋯N10	0.80 (4)	1.91 (5)	2.597 (3)	145 (4)
C6—H6*A*⋯S8	0.97	2.62	3.121 (3)	112
C15—H15⋯O18^ii^	0.93	2.48	3.404 (4)	176

Symmetry codes: (i) 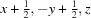; (ii) 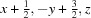.

**Table 2:** Experimental details

Crystal data	
Chemical formula	C_12_H_15_N_3_O_2_S
*M*_r_	265.33
Crystal system, space group	Orthorhombic, *P* *n* *a*2_1_
Temperature (K)	293
*a*, *b*, *c* (Å)	11.7579 (4), 15.0584 (5), 7.1103 (3)
*V* (Å^3^)	1258.92 (8)
*Z*	4
Radiation type	Mo *K*α
μ (mm^−1^)	0.26
Crystal size (mm)	0.5 × 0.2 × 0.1

Data collection	
Diffractometer	Rigaku Oxford Diffraction SuperNova, Single source at offset/far, Eos
Absorption correction	Multi-scan (*CrysAlis PRO*; Rigaku OD, 2018 ▸)
*T*_min_, *T*_max_	0.483, 1.000
No. of measured, independent and observed [*I* > 2σ(*I*)] reflections	12278, 2565, 2314
*R*_int_	0.025
(sin θ/λ)_max_ (Å^−1^)	0.625

Refinement	
*R*[*F* ^2^ > 2σ(*F* ^2^)], *wR*(*F* ^2^), *S*	0.034, 0.087, 1.07
No. of reflections	2565
No. of parameters	171
No. of restraints	1
H-atom treatment	H atoms treated by a mixture of independent and constrained refinement
Δρ_max_, Δρ_min_ (e Å^−3^)	0.17, −0.17
Absolute structure	Flack *x* determined using 938 quotients [(*I* ^+^)−(*I* ^−^)]/[(*I* ^+^)+(*I* ^−^)] (Parsons *et al.*, 2013 ▸)
Absolute structure parameter	0.02 (3)

Computer programs: *CrysAlis PRO* (Rigaku OD, 2018 ▸), *SHELXT* (Sheldrick, 2015*a* ▸), *SHELXL* (Sheldrick, 2015*b* ▸) and *OLEX2* (Dolomanov *et al.*, 2009 ▸).

## supplementary crystallographic information

### Crystal data

**Table:**

C_12_H_15_N_3_O_2_S	*D*_x_ = 1.400 Mg m^−^^3^
*M**_r_* = 265.33	Mo *K*α radiation, λ = 0.71073 Å
Orthorhombic, *P**n**a*2_1_	Cell parameters from 5842 reflections
*a* = 11.7579 (4) Å	θ = 3.2–27.3°
*b* = 15.0584 (5) Å	µ = 0.26 mm^−^^1^
*c* = 7.1103 (3) Å	*T* = 293 K
*V* = 1258.92 (8) Å^3^	Block, colourless
*Z* = 4	0.5 × 0.2 × 0.1 mm
*F*(000) = 560	

### Data collection

**Table:**

Rigaku Oxford Diffraction SuperNova, Single source at offset/far, Eos diffractometer	2565 independent reflections
Radiation source: micro-focus sealed X-ray tube, SuperNova (Mo) X-ray Source	2314 reflections with *I* > 2σ(*I*)
Mirror monochromator	*R*_int_ = 0.025
Detector resolution: 15.9631 pixels mm^-1^	θ_max_ = 26.4°, θ_min_ = 2.7°
ω scans	*h* = −14→14
Absorption correction: multi-scan (CrysAlisPro; Rigaku OD, 2018)	*k* = −18→18
*T*_min_ = 0.483, *T*_max_ = 1.000	*l* = −8→8
12278 measured reflections	

### Refinement

**Table:**

Refinement on *F*^2^	Secondary atom site location: difference Fourier map
Least-squares matrix: full	Hydrogen site location: mixed
*R*[*F*^2^ > 2σ(*F*^2^)] = 0.034	H atoms treated by a mixture of independent and constrained refinement
*wR*(*F*^2^) = 0.087	*w* = 1/[σ^2^(*F*_o_^2^) + (0.0434*P*)^2^ + 0.1653*P*] where *P* = (*F*_o_^2^ + 2*F*_c_^2^)/3
*S* = 1.07	(Δ/σ)_max_ < 0.001
2565 reflections	Δρ_max_ = 0.17 e Å^−^^3^
171 parameters	Δρ_min_ = −0.17 e Å^−^^3^
1 restraint	Absolute structure: Flack *x* determined using 938 quotients [(*I*^+^)-(*I*^-^)]/[(*I*^+^)+(*I*^-^)] (Parsons *et al.*, 2013)
Primary atom site location: dual space	Absolute structure parameter: 0.02 (3)

### Special details

**Table:**

Geometry. All esds (except the esd in the dihedral angle between two l.s. planes) are estimated using the full covariance matrix. The cell esds are taken into account individually in the estimation of esds in distances, angles and torsion angles; correlations between esds in cell parameters are only used when they are defined by crystal symmetry. An approximate (isotropic) treatment of cell esds is used for estimating esds involving l.s. planes.

### Fractional atomic coordinates and isotropic or equivalent isotropic displacement parameters (Å^2^)

**Table:**

	*x*	*y*	*z*	*U*_iso_*/*U*_eq_	
N1	0.12956 (17)	0.37994 (13)	0.5311 (5)	0.0441 (5)	
C2	0.1602 (2)	0.28850 (18)	0.4815 (5)	0.0438 (7)	
H2A	0.174911	0.254390	0.594672	0.053*	
H2B	0.228621	0.288274	0.405192	0.053*	
C3	0.0625 (3)	0.2474 (2)	0.3728 (5)	0.0552 (8)	
H3A	0.050961	0.280351	0.257136	0.066*	
H3B	0.081833	0.186795	0.339747	0.066*	
O4	−0.04027 (17)	0.24774 (14)	0.4794 (3)	0.0545 (6)	
C5	−0.0710 (2)	0.3379 (2)	0.5178 (7)	0.0615 (9)	
H5A	−0.141436	0.338728	0.588879	0.074*	
H5B	−0.084419	0.368531	0.399814	0.074*	
C6	0.0177 (3)	0.3860 (2)	0.6249 (6)	0.0597 (9)	
H6A	−0.003873	0.447934	0.636901	0.072*	
H6B	0.023218	0.361089	0.750398	0.072*	
C7	0.2028 (2)	0.44909 (15)	0.5299 (5)	0.0388 (6)	
S8	0.16206 (6)	0.55578 (4)	0.54532 (17)	0.0552 (2)	
N9	0.31482 (18)	0.42656 (14)	0.5168 (4)	0.0406 (6)	
H9	0.337 (2)	0.3724 (19)	0.521 (6)	0.044 (8)*	
N10	0.39379 (18)	0.49289 (14)	0.4965 (3)	0.0384 (6)	
C11	0.4978 (2)	0.47549 (15)	0.5317 (5)	0.0366 (5)	
H11	0.519030	0.418814	0.569994	0.044*	
C12	0.5829 (2)	0.54502 (15)	0.5115 (4)	0.0353 (6)	
C13	0.5532 (2)	0.63316 (17)	0.4666 (4)	0.0394 (6)	
C14	0.6374 (3)	0.69733 (19)	0.4546 (5)	0.0512 (8)	
H14	0.617815	0.755628	0.426000	0.061*	
C15	0.7497 (3)	0.6758 (2)	0.4845 (4)	0.0542 (8)	
H15	0.805097	0.719608	0.475521	0.065*	
C16	0.7811 (2)	0.5896 (2)	0.5277 (6)	0.0537 (7)	
H16	0.857037	0.575138	0.547403	0.064*	
C17	0.6977 (2)	0.52566 (18)	0.5408 (6)	0.0466 (6)	
H17	0.718495	0.467720	0.570242	0.056*	
O18	0.44378 (18)	0.65822 (14)	0.4336 (4)	0.0513 (6)	
H18	0.402 (3)	0.617 (3)	0.446 (6)	0.073 (13)*	

### Atomic displacement parameters (Å^2^)

**Table:**

	*U*^11^	*U*^22^	*U*^33^	*U*^12^	*U*^13^	*U*^23^
N1	0.0326 (10)	0.0409 (10)	0.0590 (14)	−0.0031 (9)	0.0038 (14)	−0.0046 (15)
C2	0.0354 (13)	0.0407 (13)	0.0551 (19)	−0.0024 (11)	0.0056 (13)	−0.0046 (13)
C3	0.0576 (18)	0.0553 (18)	0.0528 (19)	−0.0181 (15)	0.0075 (16)	−0.0038 (15)
O4	0.0436 (11)	0.0530 (11)	0.0670 (16)	−0.0148 (9)	0.0020 (11)	0.0020 (11)
C5	0.0376 (14)	0.0584 (17)	0.088 (3)	−0.0044 (13)	−0.0064 (19)	0.014 (2)
C6	0.0370 (16)	0.0545 (17)	0.088 (3)	−0.0016 (14)	0.0139 (17)	−0.0090 (18)
C7	0.0360 (12)	0.0418 (12)	0.0387 (14)	−0.0009 (10)	−0.0011 (15)	0.0015 (15)
S8	0.0481 (4)	0.0396 (3)	0.0780 (6)	0.0056 (3)	0.0010 (5)	−0.0010 (5)
N9	0.0333 (11)	0.0329 (10)	0.0556 (16)	−0.0031 (8)	0.0009 (12)	0.0040 (12)
N10	0.0336 (11)	0.0359 (10)	0.0458 (15)	−0.0050 (9)	−0.0004 (10)	0.0032 (10)
C11	0.0388 (13)	0.0329 (10)	0.0380 (13)	−0.0003 (9)	−0.0010 (14)	0.0021 (14)
C12	0.0353 (12)	0.0354 (11)	0.0353 (16)	−0.0029 (9)	0.0002 (12)	−0.0007 (12)
C13	0.0418 (14)	0.0356 (12)	0.0408 (15)	0.0001 (11)	0.0026 (12)	−0.0019 (12)
C14	0.0605 (19)	0.0351 (13)	0.0581 (19)	−0.0111 (13)	0.0005 (16)	0.0001 (14)
C15	0.0534 (17)	0.0585 (17)	0.0507 (19)	−0.0249 (15)	−0.0003 (15)	−0.0007 (15)
C16	0.0363 (14)	0.0681 (17)	0.0567 (18)	−0.0104 (13)	−0.0022 (17)	0.0016 (19)
C17	0.0389 (14)	0.0465 (13)	0.0545 (16)	−0.0001 (11)	−0.0039 (18)	0.0019 (18)
O18	0.0448 (11)	0.0349 (10)	0.0744 (15)	0.0042 (9)	−0.0019 (11)	0.0049 (10)

### Geometric parameters (Å, º)

**Table:**

N1—C2	1.466 (3)	N9—H9	0.86 (3)
N1—C6	1.478 (4)	N9—N10	1.371 (3)
N1—C7	1.351 (3)	N10—C11	1.275 (3)
C2—H2A	0.9700	C11—H11	0.9300
C2—H2B	0.9700	C11—C12	1.456 (3)
C2—C3	1.516 (4)	C12—C13	1.409 (3)
C3—H3A	0.9700	C12—C17	1.396 (4)
C3—H3B	0.9700	C13—C14	1.386 (4)
C3—O4	1.426 (3)	C13—O18	1.361 (3)
O4—C5	1.432 (4)	C14—H14	0.9300
C5—H5A	0.9700	C14—C15	1.376 (4)
C5—H5B	0.9700	C15—H15	0.9300
C5—C6	1.481 (5)	C15—C16	1.383 (4)
C6—H6A	0.9700	C16—H16	0.9300
C6—H6B	0.9700	C16—C17	1.377 (4)
C7—S8	1.680 (2)	C17—H17	0.9300
C7—N9	1.364 (3)	O18—H18	0.80 (4)
			
C2—N1—C6	112.7 (2)	N1—C7—N9	115.1 (2)
C7—N1—C2	124.4 (2)	N9—C7—S8	121.17 (18)
C7—N1—C6	121.5 (2)	C7—N9—H9	121.8 (18)
N1—C2—H2A	110.0	C7—N9—N10	118.7 (2)
N1—C2—H2B	110.0	N10—N9—H9	119.5 (18)
N1—C2—C3	108.6 (2)	C11—N10—N9	118.6 (2)
H2A—C2—H2B	108.4	N10—C11—H11	120.3
C3—C2—H2A	110.0	N10—C11—C12	119.5 (2)
C3—C2—H2B	110.0	C12—C11—H11	120.3
C2—C3—H3A	109.3	C13—C12—C11	121.9 (2)
C2—C3—H3B	109.3	C17—C12—C11	120.0 (2)
H3A—C3—H3B	107.9	C17—C12—C13	118.1 (2)
O4—C3—C2	111.7 (3)	C14—C13—C12	119.5 (3)
O4—C3—H3A	109.3	O18—C13—C12	122.3 (2)
O4—C3—H3B	109.3	O18—C13—C14	118.1 (3)
C3—O4—C5	108.6 (2)	C13—C14—H14	119.6
O4—C5—H5A	109.1	C15—C14—C13	120.7 (3)
O4—C5—H5B	109.1	C15—C14—H14	119.6
O4—C5—C6	112.6 (2)	C14—C15—H15	119.6
H5A—C5—H5B	107.8	C14—C15—C16	120.8 (3)
C6—C5—H5A	109.1	C16—C15—H15	119.6
C6—C5—H5B	109.1	C15—C16—H16	120.6
N1—C6—C5	111.4 (3)	C17—C16—C15	118.7 (3)
N1—C6—H6A	109.4	C17—C16—H16	120.6
N1—C6—H6B	109.4	C12—C17—H17	118.9
C5—C6—H6A	109.4	C16—C17—C12	122.1 (3)
C5—C6—H6B	109.4	C16—C17—H17	118.9
H6A—C6—H6B	108.0	C13—O18—H18	110 (3)
N1—C7—S8	123.73 (19)		
			
N1—C2—C3—O4	−58.7 (4)	N9—N10—C11—C12	−179.9 (3)
N1—C7—N9—N10	173.8 (3)	N10—C11—C12—C13	4.5 (5)
C2—N1—C6—C5	−50.2 (4)	N10—C11—C12—C17	−176.9 (3)
C2—N1—C7—S8	167.6 (3)	C11—C12—C13—C14	178.1 (3)
C2—N1—C7—N9	−13.0 (5)	C11—C12—C13—O18	−2.1 (4)
C2—C3—O4—C5	62.2 (4)	C11—C12—C17—C16	−178.5 (4)
C3—O4—C5—C6	−59.5 (4)	C12—C13—C14—C15	0.6 (5)
O4—C5—C6—N1	53.7 (4)	C13—C12—C17—C16	0.2 (5)
C6—N1—C2—C3	51.7 (4)	C13—C14—C15—C16	−0.2 (5)
C6—N1—C7—S8	−27.0 (5)	C14—C15—C16—C17	−0.2 (5)
C6—N1—C7—N9	152.5 (3)	C15—C16—C17—C12	0.2 (6)
C7—N1—C2—C3	−141.7 (3)	C17—C12—C13—C14	−0.6 (4)
C7—N1—C6—C5	142.7 (3)	C17—C12—C13—O18	179.3 (3)
C7—N9—N10—C11	162.4 (3)	O18—C13—C14—C15	−179.3 (3)
S8—C7—N9—N10	−6.7 (4)		

### Hydrogen-bond geometry (Å, º)

**Table:**

*D*—H···*A*	*D*—H	H···*A*	*D*···*A*	*D*—H···*A*
N9—H9···O4^i^	0.86 (3)	2.33 (3)	3.141 (3)	157 (3)
O18—H18···N10	0.80 (4)	1.91 (5)	2.597 (3)	145 (4)
C6—H6*A*···S8	0.97	2.62	3.121 (3)	112
C15—H15···O18^ii^	0.93	2.48	3.404 (4)	176

Symmetry codes: (i) *x*+1/2, −*y*+1/2, *z*; (ii) *x*+1/2, −*y*+3/2, *z*.
